# Routine data for malaria morbidity estimation in Africa: challenges and prospects

**DOI:** 10.1186/s12916-020-01593-y

**Published:** 2020-06-03

**Authors:** Victor A. Alegana, Emelda A. Okiro, Robert W. Snow

**Affiliations:** 1 Population Health Unit, Kenya Medical Research Institute - Wellcome Trust Research Programme, P.O. Box 43640, Nairobi, 00100 Kenya; 2 Geography and Environmental Science, University of Southampton, Southampton, SO17 1BJ UK; 3 Faculty of Science and Technology, Lancaster University, Lancaster, LAI 4YW UK; 4 Centre for Tropical Medicine and Global Health, Nuffield Department of Clinical Medicine, University of Oxford, Oxford, OX3 7LJ UK

**Keywords:** Malaria burden, Morbidity, Routine surveillance

## Abstract

**Background:**

The burden of malaria in sub-Saharan Africa remains challenging to measure relying on epidemiological modelling to evaluate the impact of investments and providing an in-depth analysis of progress and trends in malaria response globally.

In malaria-endemic countries of Africa, there is increasing use of routine surveillance data to define national strategic targets, estimate malaria case burdens and measure control progress to identify financing priorities. Existing research focuses mainly on the strengths of these data with less emphasis on existing challenges and opportunities presented.

**Conclusion:**

Here we define the current imperfections common to routine malaria morbidity data at national levels and offer prospects into their future use to reflect changing disease burdens.

## Background

### Malaria burden estimation

The precise burden of malaria in sub-Saharan Africa has remained elusive [1]. Infection with *Plasmodium falciparum* is a frequent event for individuals living in stable transmission areas in Africa, and not all new infections cause illness in part as a result of acquired immunity. Owing to the vagaries of malaria definitions and the ability to capture data from routine systems, the malaria community has defaulted to epidemiological models to estimate the morbid and fatal burdens of malaria [2–7]. While current burden estimation combines epidemiological modelling with aspects of routine data [6], the epidemiological models are based on sparse epidemiological surveys, a presumed understanding of the relationship between infection and disease outcome, and despite increasing mathematical complexity over time, continue to be estimated with wide margins of uncertainty [8, 9]. In the absence of empirical routine data on malaria morbidity, these models continue to be used by international agencies to prioritise malaria funding and to predict the impact of investment [10].

The National Malaria Strategic Plans (NMSPs) are developed to guide national partnerships on intervention delivery and ambition to reduce disease incidence in line with targets established by the Global Technical Strategy (GTS) for malaria [11]. In Table 1, 47 most recently available NMSPs show that targeted goals for national governments specify elimination in 14 countries, and the reduction in national case incidence in 33 countries not actively implementing elimination activities (Table 1). The ambitions of the NMSPs are articulated in terms of reducing case incidence. Furthermore, the sub-national theoretical priority setting is often represented through case incidence maps in 21 countries or based on the number of reported malaria cases from routine data (Table 1). Thirteen countries use parasite prevalence and 7 countries use climate-based maps within NMSPs to define the sub-national heterogeneity of risk. At the launch of the Roll Back Malaria initiative, 20 years ago, the use of any data to provide a strategic direction at national levels was rare. There is now at least a recognition that data should be used to inform national targets and priority setting. Whether, at the country level, data is used to provide sub-national priorities or used to measure if malaria targets are met is less clear.

**Table 1:** Country-level national malaria strategy (NMS) policy goals in sub-Saharan Africa

Country	Classification category	NMS period	National malaria strategy goal	Sub-national representation of malaria heterogeneity
Botswana	Elimination	2014–2018	Achieve zero local malaria transmission in Botswana by 2018	A and B
Cape Verde	Elimination	2014–2020	Sustainably reduce the incidence of indigenous malaria by 2016 and lay the foundations for its elimination by 2020	No malaria map
Comoros	Elimination	2017–2021	Reduce to zero cases of indigenous malaria transmission in the Union of Comoros by 2021	A
Eswatini	Elimination	2015–2020	Eliminate malaria by 2015 and achieve the WHO’s certification of elimination by 2018	B
Namibia	Elimination	2017–2022	Achieve zero local malaria cases in Namibia by 2022	A
São Tomé and Príncipe	Elimination	2017–2021	By 2021, reduce malaria incidence to 1 case per 1000 population in all São Tomé districts and 0 (0) indigenous cases in the Autonomous Region of Príncipe	B
South Africa	Elimination	2019–2023	Achieve zero local malaria transmission in South Africa by the year 2023	B
Djibouti	Pre-elimination	2013–2017	Reduce the prevalence of malaria parasite carriers from 0.64% (2008 survey) to 0% to reach zero indigenous cases by the end of 2017	D
Rwanda	Pre-elimination	2013–2020	Reduce malaria morbidity by 30% of 2015–2016 level, by 2020	A
Zanzibar	Pre-elimination	2016–2020	Detecting and responding to malaria outbreaks	B
Zimbabwe	Pre-elimination	2016–2020	Reduce malaria incidence to 5/1000 by 2020 compared to 2015 levels	A
Ethiopia	Control and elimination	2014–2020	Achieve 75% reduction in malaria cases from baseline of 2013 by 2020. Achieve falciparum malaria elimination in selected low transmission areas by 2020.	A
Somalia	Control and elimination	2016–2020	Reduce case incidence to < 1 case per 1000 in low transmission regions. Reduce case incidence by 40% in control regions	D
Zambia	Control and elimination	2017–2021	Reduce malaria incidence from 336 cases per 1000 population in 2015 to less than 5 cases per 1000 population by 2019	B
Angola	Control	2016–2020	Reduce malaria morbidity by 60% in the country by 2020 compared to the 2012 baseline.	E
Benin	Control	2017–2021	Reduce the rate of incidence of malaria by at least 25% over the 2015 rate	E
Burkina Faso	Control	2014–2017	Reduce morbidity by 75% compared to 2000	No malaria map
Burundi	Control	2018–2023	Reduce malaria morbidity by at least 60% by 2023	A and D
Cameroon	Control	2014–2018	Reduce malaria incidence from 2015 levels by 60% by 2023	E
The central African Republic	Control	2016–2020	Reduce the incidence of malaria by at least 40% in 2020 compared to 2016	E
Chad	Control	2019–2023	Reduce malaria morbidity by 75% compared to the 2015 level	A, D and E
Congo	Control	2018–2022	Reduce malaria incidence rate by 86% compared to baseline rate in 2015	No Malaria Map
Côte d’Ivoire	Control	2016–2020	Reduce the incidence of malaria by at least 40% by 2020 compared to 2015	A
The Democratic Republic of the Congo	Control	2016–2020	By 2020, reduce malaria-related morbidity by 40% compared to 2015 levels	D and E
Equatorial Guinea	Control	2016–2020	By 2020, reduce by 40% the malaria morbidity compared to the 2015 level	No malaria map
Eritrea	Control	2015–2019	Reduce malaria incidence by 50% from 2010 levels and achieve test positivity rate (TPR) below 5% in all sub-zones to shift to pre-elimination by 2017 and beyond	B and D
Gabon	Control	2018–2021	By 2021, reduce malaria-related morbidity by at least 40% compared to 2015	No malaria map
The Gambia	Control	2014–2020	Reduce malaria case incidence by at least 40% compared with 2013, by 2020	A
Ghana	Control	2014–2020	Reduce malaria morbidity burden by 75% (using 2012 as baseline) by the year 2020	D and E
Guinea	Control	2018–2022	Achieve pre-elimination by 2022 by reducing malaria morbidity by 75% compared to 2016	D and E
Guinea-Bissau	Control	2018–2022	Reduce malaria morbidity by at least 50% compared to 2015	No malaria map
Kenya	Control	2019–2023	Reduce malaria incidence and deaths by at least 75% of the 2016 levels by 2023	D
Liberia	Control	2016–2020	By 2020, reduce illnesses caused by malaria by 50% compared to MIS 2011 baseline	C and D
Madagascar	Control	2013–2017	Reduce malaria-related morbidity to less than 5% in 50% of districts and to less than 10% in other districts by the end of 2017	A, B and D
Malawi	Control	2017–2022	To reduce malaria incidence by at least 50% from a 2016 baseline of 386 per 1000 population to 193 per 1000	A
Mali	Control	2018–2022	Reduce malaria incidence by 50% compared to 2015	D
Mauritania	Control	2014–2020	Achieving the goal of eliminating malaria by 2025	B and E
Mozambique	Control	2017–2022	Reduce malaria morbidity at a national level by at least 40% compared to levels observed in 2015, by 2022	A and D
Niger	Control	2017–2021	Reduce the incidence of malaria by at least 40% by 2021 compared to 2015	No malaria map
Nigeria	Control	2014–2020	Reduce malaria burden to pre-elimination levels	D
Senegal	Control	2016–2020	Reduce the incidence of malaria by at least 75% compared to 2014	A and D
Sierra Leone	Control	2016–2020	Reduce malaria morbidity by at least 40% compared with 2015 by 2020	D
South Sudan	Control	2014–2021	Reduce the morbidity of malaria by 80% and malaria parasite prevalence by 50% compared to 2013 by the year 2020	D
Sudan	Control	2018–2020	Reduce malaria morbidity by 30% by 2020 (taking 2017 as a baseline)	D
Tanzania	Control	2014–2020	Reduce the average country malaria prevalence from 10% in 2012 to 5% in 2016 and further in 2020 to less than 1%.	D
Togo	Control	2017–2022	Reduce malaria morbidity in the general population	A
Uganda	Control	2014–2020	Reduce malaria morbidity to 30 cases per 1000 population by 2020. Reduce the malaria parasite prevalence to less than 7% by 2020.	D

For each county, the malaria vision, mission was reviewed. This table only summarises the main objective stated in the NMS. For sub-national heterogeneity, A represents the map of case incidence; B, map of malaria cases; C, map based on test positivity rate (TPR); D, map based on parasite prevalence; and E, map of climate/seasonal/ecological suitability

**Fig. 1:**
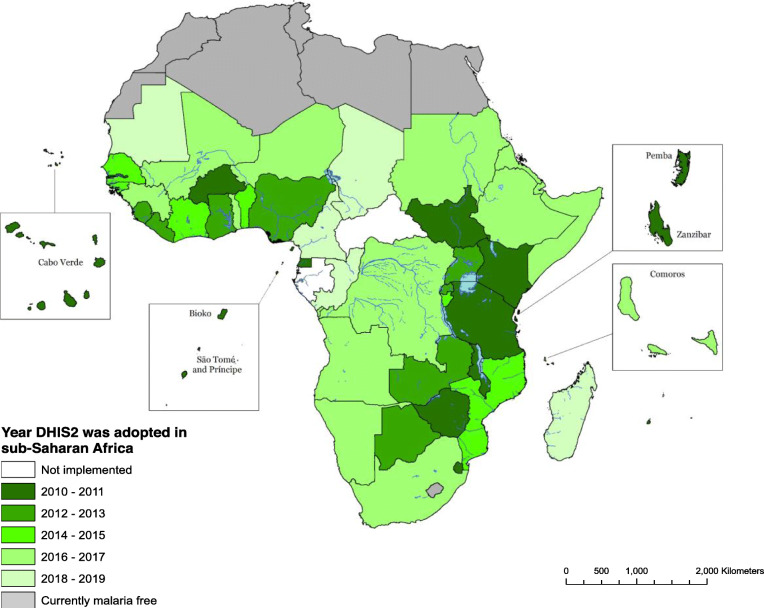
The uptake and use of District Health Information Systems (DHIS2) in Africa for routine data management. No information is available for Gabon and Central Africa Republic. For these countries, it is assumed piloting is underway or planned

The increasing use of nationally owned surveillance data to define malaria burdens has likely emerged because of three key important initiatives. Firstly, the ability to define malaria-specific morbidity presenting to the health services has been improved substantially with the universal acceptance across Africa of the Test. Treat. Track (T3) initiative [12], facilitated by the innovation in point-of-care, malaria rapid diagnostic tests (mRDTs). Between 2010 and 2018, over 1 billion mRDTs have been performed in Africa [13]. Secondly, there has been a recognition that routine data should form the basis of improved malaria control. In 2015, the GTS was developed and transformed malaria surveillance into a core malaria intervention. Finally, across Africa since 2010, there has been an unprecedented harmonisation of electronic health data management platforms, especially the District Health Information Systems (DHIS2) [14, 15] (Fig. 1). This adaptable electronic data platform has enabled malaria programmes to work with national health information partners to ensure there are malaria dash-boards that capture data, from public and private health sectors.

**Fig. 2:**
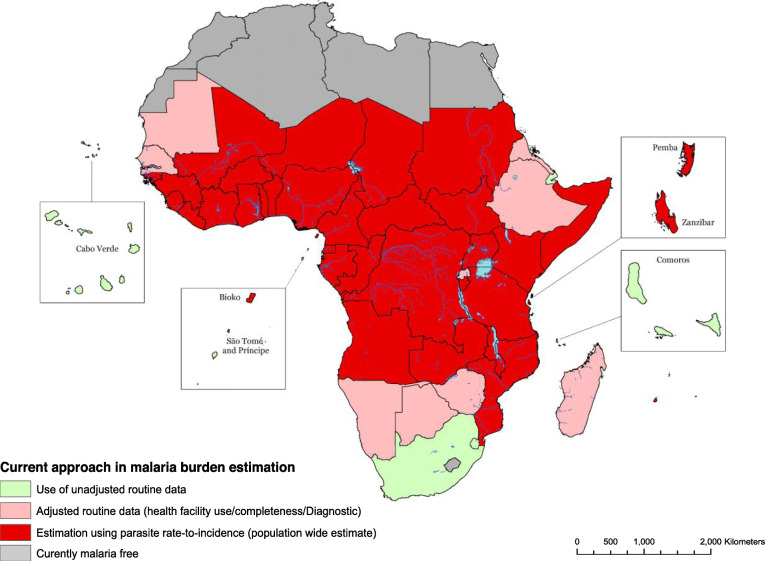
Map of sub-Saharan Africa showing the current methodologies used to estimated malaria case burden based on the World Health Organization (WHO) report [16]. Category 1 is used in countries with high-quality surveillance systems and near elimination. Thus, routine data is used without adjustments. For category 2, routine data are adjusted for test positivity rate, public health sector reporting rate, fever treatment-seeking rate and rates of not seeking treatment. For category 3, parasite rate-to-incidence conversion is used

Clearly, countries in Africa aiming to eliminate the parasite nationwide, or within defined geographic areas, are required to identify all cases of the disease and new infections. Countries in this category include Cape Verde, Comoros, São Tomé and *Príncipe*, South Africa and Eswatini (Table 1; Fig. 2), where the WHO uses the actual cases of malaria reported as the definitive number of new malaria cases each year. The challenges associated with surveillance in identifying every new infection for elimination have been considered elsewhere [9, 17, 18]. With improvements in detection, treatment and reporting, several other countries provide routine data to the WHO for their World Malaria Report (WMR), including Botswana, Eritrea, Ethiopia, The Gambia, Madagascar, Mauritania, Namibia, Rwanda, Senegal and Zimbabwe (Fig. 2). Case data in these second-tier countries are adjusted to reflect cases that might have been missed from formal reporting systems [13]. However, for 30 countries in SSA, the WHO [13] uses modelled predictions from a composite of interpolated, modelled parasite infection prevalence surveys undertaken infrequently, and transformed to case incidence using a modelled non-linear relationship between parasite prevalence and active case detection from 30 epidemiological studies undertaken between 10 and 20 years ago [19] (Fig. 2). Outside of Africa, routine data reported by national malaria control programmes are almost universally used as a direct estimate of the clinical burden per country. One obvious ambition of the WHO’s GTS is that all countries worldwide have robust, reliable and timely surveillance, avoiding reliance upon uncertain modelled estimates of malaria disease burden.

## Main text

### Components of reliable routine surveillance for malaria morbidity burden estimation

Surveillance involves the continuous collection and use of data to inform health policy and decision-making. However, there are challenges for Routine Health Information System (RHIS) related to the technical processes (e.g. data flow, recording forms, system-related), organisational (e.g. resources, structures, information cultures) and user behavioural (e.g. health worker knowledge, skills, attitudes). Reviews on the technical challenges and improvements in RHIS are presented elsewhere [14, 20, 21]. Many of these equally apply to malaria; however, there are several aspects of malaria burden estimation through RHIS that require specific consideration.

**Fig. 3:**
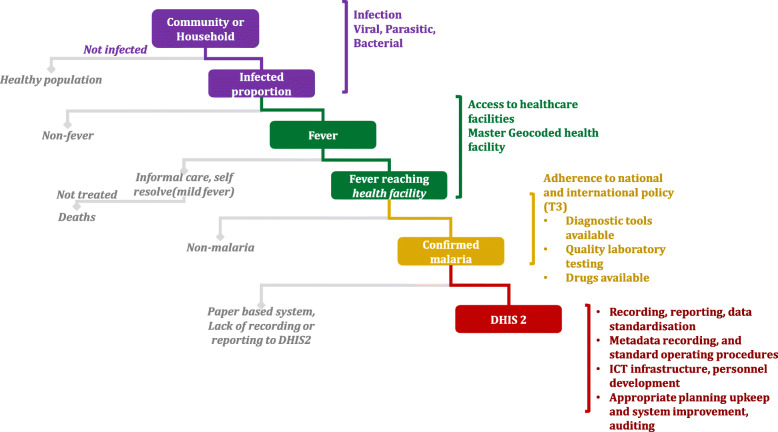
Ideal malaria routine data flow. The ideal system would require all fever cases occurring at community-level use health facilities and that a complete geo-coded master health facility list. Fever cases presenting at health facilities are then tested for malaria under the Test.Treat.Track (T3) initiative. Thus, appropriate diagnostics or laboratory tools should be available at the health facility, the quality of laboratory testing should be highest, there should be no drug stock-outs and the treatment of fever case should be based on the national guidelines at the health facility. Finally, all confirmed malaria cases at the health facility should be recorded accurately and reported promptly to the national surveillance system such as DHIS2

The use of routine data for malaria morbidity estimation requires an understanding of the denominator population from which the cases originate, completeness and demographics of the number of reported malaria cases, and the uncertainties or biases associated with these quantities. Ideally, all fevers that could be malaria occurring within a community must reach a facility where parasitological testing is provided, and all these events are accurately recorded and stored within a real-time electronic data capture system, such as DHIS2 (Fig. 3). This is rarely the case in Africa settings, and until this ideal is reached, there is a need to estimate the numbers of fevers not reaching diagnostic centres, the fraction tested, and of those who do not reach testing centres or those untested, the presumed fraction positive. The variance from the ideal to reality can be a result of multiple factors. These factors and components of RHIS are discussed below and demonstrated in Fig. 3.

#### The denominator population

One starting point is an understating of population denominator from which malaria cases arise. Fine-scale census data is often not available or accessible to NMPs. Population censuses are conducted every 10 years and, in some countries (the Central African Republic, the Democratic Republic of the Congo, Eritrea, Somalia and Madagascar), the last census was conducted over two decades ago [22, 23]. In countries with a recent census, data is not always accessible at granular age or fine-spatial scales. This highlights a broader interoperability issue between government ministries and departments. Timely and fine-scale census data is fundamental to understanding health access, health service catchments and sub-national disease burden. Consequently, coarse-scale census data is used to provide open-access population-density surfaces, disaggregated to a fine-scale using weighted dasymetric mapping [24–29]. These include 1 × 1 km gridded population surfaces produced by Worldpop [30], Gridded Population of the World (GPW) [31], LandScan [32] and Facebook [33], with Worldpop being the most frequently used in malaria burden estimation [5–7, 13]. Modelling of imperfect spatial and temporal census data comes with uncertainty [34, 35] and cannot replace empirical local, fine-scale population data if these were made more accessible. There are new innovative methods of mapping population combining social media platforms with satellite remote sensing via machine learning methods [36], or triangulating data from human settlements with mobile phones [37]. Integrating these novel methods of human population and settlement locations into more efficient definitions of health facility catchments should be encouraged.

Malaria-morbidity-specific catchments are important in interpreting facility-level enumerations of the case burden and identifying those populations marginalised from formal health services [38–41]. Ideally, the definition or the demarcation of a health facility catchment should be based on choices made by patients seeking care at the health facility rather than solely on proximity (distance) [42–44]. Patient choice of health services depends on many individual-level factors described earlier (location and behaviour) and system factors such as competition between accessible service providers. Improvement in catchment demarcation could, therefore, be improved by integrating these demand and supply factors, from DHIS2, with community-level factors.

#### A Master Health Facility List

In defining catchment population, an important aspect is whether the DHIS2 represents the universe of all healthcare providers within a country. Censuses of healthcare providers are increasing in scope and coverage across Africa, through the Master Health Facility List (MHFL) initiative [45]. MHFL has been established and updated in 11 countries (Burundi, Botswana, The Democratic Republic of the Congo, Malawi, Namibia, Nigeria Rwanda, Kenya, Swaziland and Zimbabwe). While this should form the basis of examining the completeness of reporting from formal health provider sectors, many other countries do not have an updated and available census of providers, and fewer are geo-referenced for use in a more granular form to examine sub-national heterogeneity or understand the completeness in fever diagnosis [46].

#### Variation in fever treatment-seeking behaviour

Malaria fevers among semi-immune populations can be self-limiting, and patients may not seek treatment. Sources of general fever treatment are manifold. Individuals and caretakers seek fever treatment from medicines available at home, shops, drug vendors, private informal healthcare providers and formal health sector, and polypharmacy is common [47, 48]. For example, the latest WMR states that approximately 36% (interquartile range [IQR] 28–45%) of children in SSA with a fever in the last 2 weeks did not seek treatment [13]. Data has been used to map the variation in fever treatment-seeking among children across Africa using household survey data on the actions taken for fevers reported by carers over the last 14 days [49–51]. These household surveys rarely capture the complexity of first, second, or third sources of treatment; cannot define what treatments might be sought after the interview; and do not capture febrile populations older than 5 years.

Patient choice depends on different factors such as the distance, social, cultural, costs, and attractive properties of the health facility [52–58]. Referrals from one sector to a higher-level facility are complex; patients frequently by-pass their nearest service provider for several reasons [59, 60]. Surprisingly, little is known about the choices made for treatment by febrile children, including the contextual nature of choice (disease and healthcare quality perceptions or geographic access) [44, 61].

There is limited information on malaria patient groups outside of childhood. The risks of fevers associated with malaria infection, treatment-seeking, diagnostic use and documentation among non-pregnant adults in Africa are rarely described. Aggregated routine data is often reported by age groups above and below 5 years, limiting the ability to understand the epidemiology of malaria morbidity in the entire community by age [62]. The highest burden of severe malaria and malaria mortality is concentrated among young children. However, infection and mild clinical disease continue to pose a burden on adolescents [63] and less so in non-pregnant adults [64, 65].

There is a need to understand treatment choices to define malaria fevers likely to be missed through routine data. This will require more in-depth quantitative survey questions combined with qualitative methods across all age groups.

#### Malaria testing

Not all fevers reaching the health facility are tested for the presence of malaria parasites [66]. For decades, healthcare providers in malaria-endemic areas treated all fevers as malaria presumptively [67–70]. In 2011, the international malaria case-management guidelines were changed to improve parasitological testing and treatment adherence to malaria test results [12, 71]. This has now been adopted widely across SSA. According to the latest WMR, over 66% (IQR 49–75%) of childhood fevers presenting to a formal healthcare providers in 20 SSA countries were reported to have been subjected to a parasitological test [13]. This remains a long way from universal testing of all fevers presenting to health facilities with a capability of providing this service. The variation between and within country in testing rates can result from inadequate training and lack of supervision of healthcare workers [72–74], shortages and stock-outs of equipment and mRDTs [75, 76], and patient-level factors [77, 78]. These are health system issues that are surmountable by improving in-service training, stock management and logistics. Importantly, the RHIS can identify these failings to specific health facilities, becoming a self-regulating district supervisory tool.

However, universal parasitological testing is more challenging among those who seek treatment in the informal private sector or at home. Efforts to roll out mRDTs through community healthcare workers [79–83] or informal retailers [84–86] are underway, and these are currently reported to the DHIS2 at health facilities where the community health worker is attached or through mobile systems. Ensuring quality diagnosis and treatment as close to the home as possible is critical to ensuring appropriate treatment. However, for morbidity burden estimation and surveillance, innovation is required to ensure all cases are documented and tracked effectively.

Presently, malaria routine data that is used by the WHO for burden estimation presumes that the fraction of parasite-positive fevers in the formal health sector are the same as those who remain untreated or treated in the informal sector. Few empirical surveys have examined infection prevalence in childhood fevers in the community versus those reaching facilities [39, 65, 87, 88], or those seen at accredited drug stores versus formal health facilities [89]. This represents a data gap and needs further exploration, across all age groups, to validate corrections made to fever incidence that does not reach facilities.

With more empirical parametrisation of how malaria fevers are treated and choices at a household level, more formal statistical approaches might be applied to malaria treatment-seeking behaviour data. This can then be combined with DHIS2 to improve the understanding of events missed. Understanding the contextual factors that influence choices, including distance to services, seasonal influences on access, service quality, service costs and poverty could be integrated within geo-statistical platforms that could accommodate multiple levels of predictive information that would not assume all treatment-seeking is uniform within a single country. Examples of how individuals interact with health systems have been developed using probit behavioural models that incorporate latent variables, for example, Item Response Theory [90–93], that also allow the quantification of unobserved individual-level traits influencing behavioural outcomes.

Currently, mRDTs are replacing microscopy as the diagnostic of choice. The most commonly used mRDTs detect antigens produced by *Plasmodium* parasites circulating in the blood such as the *Plasmodium falciparum* histidine-rich protein-2 (PfHPR2) or *Plasmodium falciparum* histidine-rich protein-3 (PfHPR3) [94]. Evolutionary fitness to avoid detection has resulted in deletions in the parasite to PfHPR2/3 in SSA [95–97]*.* The extent or distribution of this phenomenon in other settings in Africa is not yet clear. The current recommendation is using mRDTs that do not exclusively rely on PfHPR2/3 in areas where PfHPR2/3 deletions are found prevalent [98]. This will require a dual approach to surveillance of PfHRP2/3 deletions and innovation in new mRDTs.

#### Coverage of routine data for decision-making in DHIS2

While there has been rapid adoption of DHIS2 across Africa (Fig. 1), barriers exist related to data access, data quality, transparency, use at international and national levels [99] and the existence, in some countries, of multiple data systems operating in parallel. Operationally, routine data systems use multiple registers for data capture at the health facility level. These are typically located in different departments such as the outpatient departments, the inpatient department, antenatal clinics and the laboratory. The variation in data capture and multiple recording contribute to inconsistencies and delays while transferring data from registers to aggregate (facility-month) malaria cases and subsequent reporting in DHIS2 [100–104].

Incomplete reporting of routine data is common across all surveillance systems. This might include facilities never reporting, facilities missing some months of data, and incomplete reporting of data elements. The former requires a complete inventory of facilities. Monthly data might be available at the health facility level or aggregated across time and districts. Aggregated data present challenges in understanding the true completeness and masks data quality issues at various service delivery points at the health facility level [105]. When facility-level data exhibits missingness, then statistical imputation techniques can be employed: for example, using moving averages within the longer-term data at that facility [106, 107] or including neighbouring facility data and information on seasonality through model-based framework [108–111]. While data incompleteness requires health system interventions and quality assurance methods, dealing with incomplete data remains an academic exercise. National Malaria Programmes (NMPs) require skills to understand the impact and statistical consequences of incomplete data and training in simplified tools to improve sub-national disease burden estimation. Developing capacity within NMPs for effective analysis (spatial or non-spatial analysis) of routine health facility data, visualisation (e.g. using Geographic Information Systems) and their interpretation to promote a culture of evidence-driven decision-making requires long-term, sustainable investment [112, 113], circumventing the need for externally driven analysis of national data.

### Other uses of routine data

Routine data are not only used by national malaria programmes to define disease burden. Other routine metrics provided by DHIS2 include fever test positivity rates (TPR) which have historically been used to define malaria stratification to target resources for elimination [114, 115]. Tanzania provides recent examples of using the routine DHIS2 TPR data for sub-national stratification for fevers [116, 117] and pregnant women attending antenatal clinics [118, 119]. Routine data also provides the bedrock for commodity supplies, drugs, diagnostics and prevention (long-lasting treated nets, intermittent presumptive treatment). While less critically dependent on the definition of the populations they serve [9, 40], many of the elements of care-seeking, infected populations and those missed by routine data apply equally to the representativeness of TPR and those marginalised from health services.

## Conclusion

The GTS has ambitious long-term goals, including elimination. Surveillance is considered as a core intervention and a third pillar for the GTS. However, there continues to remain a focus on what commodities (including their costs) are required for disease treatment and prevention, and less on how to improve disease burden estimation at national levels. Improving burden estimation is fundamental for efficient allocation, use of resources and examining whether they have the desired impact on disease burden. The *High Burden to High Impact: A Targeted Malaria Response* [120] has begun to introduce the notion that using data to inform strategic investment is central to maximising impact. Data remain imperfect; however, with a more detailed understanding of their representativeness, missingness and epidemiological context, these data can replace modelled estimations of morbidity anchored in parasite prevalence. At the very least, data from routine reporting is a continuous measure, providing information every month of every year, unlike underpowered parasite prevalence surveys undertaken every 3–5 years [121–123]. Some of the data gaps in improving our understanding of the precision of routine malaria data are summarised in Table 2.

**Table 2:** Outstanding questions and data gaps

• Improving access to national data on fine resolution census and meteorological data • Explore new methods of defining local population denominators and catchments • Improving geo-coded inventories of health service providers • Improved understanding of fever incidence, infection risk and treatment-seeking patterns across all age groups and genders, including better structured quantitative and qualitative methodologies • Developing tools for tracking quality of data in routine data systems • Surveillance for PfHRP2/3 deletions • Building long-term, sustainable capacity in national malaria programmes (NMPs) to understand, interrogate, display and interpolate routine malaria data	

Not considered in this paper is that there is an equivalent need to examine how we define malaria mortality burdens. Measuring the achievement of zero malaria deaths requires a parallel interrogation of data systems, including the veracity of cause of death attribution and improving civil registration [124, 125], not considered here. The modelled estimation of malaria mortality is more complex [5, 126, 127], more uncertain [128, 129] and less well represented by national death registration [130–132].

If the GTS is to succeed, it should be linked to investment in routine malaria surveillance, not limited to those countries aiming for elimination, but all countries across SSA. As more countries improve their routine morbidity surveillance, and the map shown in Fig. 2 changes, new estimates of the malaria burden in Africa will emerge. The challenge is then to persuade the international community that these new estimates will not indicate a rise or fall in malaria burden, but an improvement in estimation. A sensible metric of success for the GTS would be a national ability to define its own sub-national malaria morbidity burden.

## Data Availability

No data analysis was conducted using primary or secondary data.
